# Musculoskeletal Magazine Advertising Focuses on White Individuals and Overlooks Minority Consumers

**DOI:** 10.3390/jmahp13010004

**Published:** 2025-02-04

**Authors:** Wei Shao Tung, Kelsey A. Rankin, Robert John Oris, Adithi Wijesekera, Daniel H. Wiznia

**Affiliations:** 1 Department of Orthopaedics and Rehabilitation, Yale University, New Haven, CT 06510, USA; adithiwij@gmail.com (A.W.); daniel.wiznia@yale.edu (D.H.W.); 2 Department of General Surgery, University of California San Francisco, San Francisco, CA 94143, USA; kelsey.rankin@ucsf.edu; 3 Feinberg School of Medicine, Northwestern University, Chicago, IL 60611, USA; orisrj@gmail.com

**Keywords:** racial disparities, direct-to-consumer advertising, musculoskeletal health

## Abstract

Introduction: Demographic disparities in musculoskeletal (MSK) health exist in the US. Racial representation in advertising has been shown to influence consumer behavior and buying patterns. Direct-to-consumer advertising that does not target a racially diverse audience may exacerbate MSK disparities by failing to reach minorities. We explore the hypothesis that minorities are underrepresented in direct-to-consumer MSK advertisements in this cross-sectional analysis. Methods: Using magazines from four databases, eight health-related magazine types were selected and advertisement categories were established. Racial distribution was analyzed using Pearson’s Chi-squared and Chi-squared tests. Fisher’s Exact test was used when >20% of cells had expected frequencies <5. Significance was set at α = 0.05. Results: Of the advertisements featuring at least one model, 68.5% featured a white-presenting model, followed by 17.6% with a black model. Further, 92.7% of advertisements were monoethnic or monoracial with an overrepresentation of white models (*p* < 0.001). Black models were overrepresented as athletes (*p* < 0.001) and underrepresented in advertisements for pain relief (*p* < 0.001). Hispanic/Latinx and Asian models were underrepresented across all advertisement categories (*p* < 0.001). Discussion: The causes of musculoskeletal health disparities are multifactorial. One potential influence is adjacent industries such as MSK health-related advertisements. When controlling for US population demographics, white models were overrepresented and minority race models were underrepresented, demonstrating racioethnic disparities in MSK advertising. Improving the racial and ethnic diversity of models within MSK advertisements may serve to improve patient perceptions of orthopaedic products and services and improve MSK disparities.

## 1. Introduction

Racial and ethnic disparities in musculoskeletal (MSK) health exist in a multitude of forms. Racioethnic differences can serve to determine a person’s prognosis for the development of conditions such osteoarthritis or osteoporosis, without considering environmental factors or inherited disorders [1,2,3]. Beyond this, more profound differences in MSK health emerge due to socioeconomic and geographic disparities; however, these factors are often interrelated and cannot be considered without their relation to racioethnic inequalities [4,5,6]. Social determinants of health have been found to disproportionately impact minority racial groups.

Direct-to-consumer advertising, which represents an important means of exposure to health-promoting products, may exacerbate inequality in access to musculoskeletal health if the advertisements do not target a diverse population of consumers [7]. Historically, African Americans and non-white Hispanics have been underrepresented in all types of advertising in the US, and although recent representations of African Americans have increased, they are skewed towards being depicted as athletes or musicians, feeding into historical stereotypes [8]. Racial representation within direct-to-consumer advertising can influence an individual’s purchasing decision if the consumer identifies more closely with the race and/or ethnicity of the model in an advertisement [9,10,11].

With expanding accessibility to magazines through print and online platforms, it becomes important to review the diversity in MSK health magazine advertisements and determine how these marketing practices relate to the accessibility of health-promoting products and care and to the patterns of health seen in the racioethnically diverse population in the US. Orthopaedic surgeons should be aware of how this form of media affects health literacy and consumerism within their patient population. While the interrelationship between advertisements and health have been explored extensively in other public health concerns such as smoking, there is a paucity of information on MSK conditions [12,13]. Particularly, the extent to which advertisements that promote MSK health are aimed at minority populations is currently unknown. Therefore, this study aims to examine whether racioethnic disparities exist in MSK magazine advertising, to support a better understanding of current MSK product marketing practices. We hypothesize that racioethnic diversity in MSK advertising will not be representative of the US population.

## 2. Methods

### 2.1. Study Design

A cross-sectional analysis was undertaken using MSK health advertisements collected from magazines in digital and print formats. This study design has previously been utilized within the literature [14,15,16]. We defined an MSK health advertisement as any advertisement whose product or service would benefit an individual’s orthopaedic health (skeletal, muscular, or nutritional). The following databases were used to obtain magazine content: RBdigital, ZINIO magazine database, *Magzter,* and Amazon.

The principal investigator (DW) and one researcher (AW) worked jointly to select eight magazine genres catering to the general interests of different populations within the US. Data were obtained from the following magazine types: women’s health, men’s health, food/dietary health, elderly health, sports health, general lifestyle, African American health, and Hispanic health. Each magazine genre was populated with three different magazines, with a total of 24 distinct magazines included for evaluation. A June 2019 industry whitepaper from the Alliance of Audited Media was utilized to determine magazines with the greatest subscription counts [17]. The magazines chosen for each type were based on the highest number of subscriptions and accessibility to one complete year’s worth of issues from the above databases. A complete list of magazine titles for each magazine type is included in Appendix A.

One year’s worth of magazines was examined, with the primary timeframe being January–December 2019. However, within the four magazine sources that were utilized, not all magazines were available for the complete year of 2019. When a singular issue was not available for 2019, the missing magazine issue was supplemented with an issue from the corresponding month in 2020. The majority of the magazines had a monthly publishing frequency; however, the *Black EOE Journal* published tri-annually; *Runner’s World* and *Taste of Home* published bimonthly; *The New Yorker*, *Woman’s World*, *Vanidades Mexico* and *Sports’ Illustrated* published weekly; and *Saber Vivir Ar* published biweekly. Although these magazines did not follow a monthly publishing schedule, they were included to maintain consistency in evaluating magazines with the highest subscriber count. For the magazines with weekly, biweekly, and fortnightly issues, the first issue of the month was chosen.

### 2.2. Data Collection

The following information was recorded for each issue: magazine title, magazine type, issue, page number the advertisement was found on, advertisement category, product name, size of the advertisement, description of the model(s) in the advertisement if applicable (including number of models, gender, age, and race of model(s)), presence of a celebrity, magazine frequency, and number of monthly subscriptions. The advertising categories were recorded as exercise, fitness/exercise clothing, fitness/exercise equipment, health food, pain relief, supplements, diet, health insurance, weight loss program, sports drink, medical establishment (diagnostic center), medical procedure, or medical equipment. If a certain advertisement product overlapped between two of the above advertisement categories, the category that was primarily featured in the advertisement was chosen.

The 24 magazines were each reviewed independently by two individuals (AW, RO). If consensus was not reached for a particular item after discussion, a third examiner (DW) was utilized to reach a final determination.

### 2.3. US Demographics

US racial demographics retrieved from the US Census Bureau 2021 Census Quick Facts were used to control for racial variation in candidate analyses [18]. Racial breakdowns were as follows: non-Hispanic White: 60.1%; non-Hispanic African American: 13.4%; Hispanic: 18.5%; Asian: 5.9%; Mixed race: 2.8%.

### 2.4. Statistical Analyses

Categorical variables were analyzed using Pearson’s Chi-squared tests. When >20% of cells had expected frequencies <5, Fisher’s Exact test was used. For intra-advertisement categories and intra-magazine type analyses, unknown race was removed, and Chi-squared Goodness of Fit tests were used. Goodness of Fit analyses used the distribution of the aforementioned US 2020 Census racial demographics. To minimize potential bias, only analyses with *n* ≥ 10 were reported and considered. Statistical analyses were performed using Stata v.16.1 (StataCorp, College Station, TX, USA), and graphs were created using GraphPad Prism 7.0 (GraphPad Software, Boston, MA, USA). The significance level was set at α = 0.05.

## 3. Results

### 3.1. Racial Demographics of Models in MSK Advertisements

A total of 1617 MSK advertisements were identified. No models were identified in 832 (51.5%) advertisements. Of the 785 ads with at least one model, 538 (68.5%) featured a white-presenting model, 138 (17.6%) featured a black model, 22 (2.8%) featured a Hispanic model, 10 (1.3%) featured an Asian model, and 77 (9.8%) featured a model of unknown race or ethnicity (Table 1). This result deviates significantly from US demographics (*p* < 0.001). There were 57 (7.3%) advertisements that featured multiple models of different races or ethnicities, while 728 (92.7%) did not (Table 1). Overall, white models were featured significantly more frequently than African American and Hispanic models (both *p* < 0.001).

### 3.2. Racial Demographics of Models by MSK Advertisement Category

Significant differences in race/ethnicity were observed by MSK ad category (*p* < 0.001) (Table 2). White models were significantly overrepresented in all categories apart from sports drink and health insurance advertisements, where they were underrepresented (all *p* < 0.001, medical equipment *p* = 0.037) (Table 3). African American models were overrepresented in health food, sports drinks, and fitness/exercise clothing, but were underrepresented across ads for pain relief and supplements (Table 3). Hispanic models were underrepresented across all ad categories (Table 3). Asian models had little to no representation and were underrepresented across all categories (Table 3).

## 4. Discussion

This is the first study to investigate racioethnic representation in direct-to-consumer advertising relating to MSK health. We found that of the advertisements displaying at least 1 person, 68.5% featured a white-presenting model, while the next highest represented demographic in MSK advertisements was African Americans (17.6%). There was a white predominance in almost every advertisement category that was evaluated, other than health insurance, weight loss program, sports drink, and medical establishment ads (*p* < 0.001). We found that African American models were overrepresented as athletes. In addition, there was limited representation of Hispanic and Asian models. This underrepresentation of diverse models in MSK advertising adds to a register of racial disparities and biases that already exist in MSK health and orthopaedic surgery [19,20,21,22].

Overall, racial representation is unbalanced across all MSK advertisements. We found that white models were represented significantly more than all other races, and that this representation proportionately deviated from US demographics. Despite white populations having lower rates of obesity, osteoarthritis, and overall better general health, white individuals were the most targeted audience for MSK health-promoting products, even when adjusting for US racial demographics [3,23,24,25,26]. We also found that white models were overrepresented in all ad categories, apart from sports drink and health insurance advertisements, where they were underrepresented.

African American models were overrepresented in sports drink advertisements and in sports health magazines. This reinforces extant literature, demonstrating the skewed representation of African Americans as athletes [8]. This narrow portrayal may reduce the target audience for MSK advertisements in the African American community. Additionally, we found that African Americans were underrepresented across pain relief advertisements. Racial disparities in pain management advertising are concerning, given healthcare’s historical and current underdiagnoses of pain and treatment biases in African Americans [27,28]. In contrast, health food and health insurance were two ad categories in which African Americans were over-represented, and these may potentially have a positive impact in addressing African American healthcare disparities. As African American and Hispanic individuals have lower rates of health insurance coverage than non-Hispanic White individuals, this overrepresentation in insurance advertisements can be seen to be appropriately targeting a population in need, and as a strategy that can be emulated in other categories [29].

Significant racial and ethnic disparities exist in MSK health, as evidenced by differences in the rates of obesity, hip and knee osteoarthritis, severe joint pain, lost quality-adjusted life years, and clinical and patient-reported outcomes [23,24,25,26,30,31]. Direct-to-consumer advertising is highly influential in minority communities, and race portrayal in advertising has been shown to affect customer perception, favorability, and buying patterns [9,10,11,32,33]. In the US, it has been demonstrated that African Americans and Hispanics are underrepresented in health-promoting magazine advertisements [8,15,33]. Moreover, even magazines aimed at African American and Hispanic populations have been shown to display significantly fewer health-promoting advertisements and more health diminishing advertisements—both of which may have consequences on MSK health. If the representation of racioethnically diverse models continues to be stereotypical, then the positive influence of health-promoting advertisements will only ever reach those who can relate to the portrayed, effectively eliminating any diverse representation. A shift in representation could be an important mechanism to reduce disparities in MSK health.

Companies have an obligation to shareholders to be profitable, and diversifying advertisements to increase racial representation will serve to expand product exposure and access a largely untapped consumer population. One potential avenue to increase diversity includes encouraging companies and their advertisers who did not utilize models to include racially diverse models. For example, more than half (832/1617; 51.5%) of MSK advertisements in this study did not have any model featured or displayed. This represents a straightforward and actionable opportunity in which current ads without models could be revised to include diverse models. Other alternatives are to increase the diversity of models within companies that utilized a disproportionate number of white models for ads or by depicting multiple models per advertisement.

Our study is limited by the difficulty and subjective nature of identifying models by race/ethnicity. For example, differentiating between hair color, facial features, and skin color can sometimes be ambiguous. However, there was an attempt to mitigate any potential impact of this ambiguity by utilizing two independent racially diverse reviewers and adjudicating disagreements with a third reviewer. Additionally, this subjectivity in identifying models by race/ethnicity is also present when a viewer is exposed to an advertisement. Future research on this topic can explore gender disparities, as well as the political implications of advertising practices in MSK health.

## 5. Conclusions

Of the advertisements in this study featuring at least one model, 68.5% featured a white-presenting model, followed by 17.6% with a black model. Our study demonstrates that the current racioethnic diversity in MSK advertising is not representative of the US population. Black models were overrepresented as athletes and underrepresented in advertisements for pain relief, highlighting historical stereotypes. Hispanic/Latinx and Asian models were underrepresented across all advertisement categories. Direct-to-consumer advertisements in magazines are unique in their potential ability to persuade readers to take better proactive care of their MSK health. Improving the racial and ethnic diversity of models within MSK advertisements may serve as an approach to closing the gap in MSK disparities.

## Figures and Tables

**Table 1:** Magazine and ad characteristics.

Variable	Number MSK ads Across 3 Magazines/Type; n (%)
**Magazine type**	
Senior Health	131 (8.11%)
Women’s Health	352 (21.78%)
Sports Health	401 (24.81%)
Men’s Health	355 (21.97%)
Food/Dietary Health	85 (5.26%)
General Lifestyle	211 (13.06%)
African American Health	12 (0.74%)
Hispanic Health	69 (4.27%)
**Ad category**	
Exercise	173 (10.71%)
Fitness/Exercise Clothing	250 (15.47%)
Fitness/Exercise Equipment	237 (14.67%)
Health Food	264 (16.34%)
Pain Relief	150 (9.28%)
Supplements	225 (13.92%)
Diet	199 (12.31%)
Health Insurance	19 (1.18%)
Weight Loss Program	13 (0.80%)
Sports Drink	62 (3.84%)
Medical Establishment	6 (0.37%)
Medical Procedure	2 (0.12%)
Medical Equipment	16 (0.99%)
**Featured model**	
Model	785 (48.55%)
No model	832 (51.45%)
**Multiple races in ad**	
Yes	57 (3.53%)
No	1560 (96.47%)
**Different genders in ad**	
Yes	133 (8.23%)
No	1483 (91.71%)
Unknown	1 (0.06%)
**Race of model**	
Black	138 (17.58%)
Hispanic	22 (2.80%)
Asian	10 (1.27%)
White	538 (68.54%)
Unknown or N/A	77 (9.81%)
**Featured gender**	
Female	434 (55.29%)
Male	287 (36.56%)
Unknown or N/A	1 (0.13%)
Both	63 (8.03%)
**Featured gender and race**	
White Men	196 (11.88%)
White Women	313 (18.97%)
Black Men	50 (3.03%)
Black Women	64 (3.88%)
Hispanic Men	12 (0.73%)
Hispanic Women	6 (0.36%)
Asian Men	4 (0.24%)
Asian Women	5 (0.30%)
Unknown or none	1000 (60.61%)

**Table 2:** Differences in race of model by MSK ad category.

Variable	n (%)					*p*-Value
	White	Hispanic	Asian	Black	Unknown	
Ad Category						
Exercise	93 (67.9%)	10 (7.3%)	2 (1.5%)	17 (12.4%)	15 (10.9%)	**<0.001**
Fitness/Exercise Clothing	97 (72.9%)	2 (2.3%)	3 (2.3%)	21 (15.8%)	10 (7.6%)	
Fitness/Exercise Equipment	67 (75.3%)	2 (2.3%)	3 (3.4%)	9 (10.1%)	8 (9.0%)	
Health Food	49 (69.0%)	0 (0.0%)	0 (0.0%)	17 (23.9%)	1 (7.0%)	
Pain Relief	53 (85.5%)	0 (0.0%)	1 (1.6%)	4 (6.5%)	4 (6.4%)	
Supplement	96 (81.4%)	2 (1.7%)	1 (0.9%)	7 (5.9%)	12 (10.2%)	
Diet	61 (78.2%)	1 (1.3%)	0 (0.0%)	8 (10.3%)	8 (10.3%)	
Health Insurance	7 (41.2%)	0 (0.0%)	0 (0.0%)	10 (58.8%)	0 (0.0%)	
Weight Loss Program	2 (25.0%)	0 (0.0%)	0 (0.0%)	6 (75.0%)	0 (0.0%)	
Sports Drink	0 (0.0%)	0 (0.0%)	0 (0.0%)	39 (72.2%)	15 (27.8%)	
Medical Establishment	0 (0.0%)	5 (100.0%)	0 (0.0%)	0 (0.0%)	0 (0.0%)	
Procedure	2 (100.0%)	0 (0.0%)	0 (0.0%)	0 (0.0%)	0 (0.0%)	
Medical Equipment	10 (100.0%)	0 (0.0%)	0 (0.0%)	0 (0.0%)	0 (0.0%)	

Bold indicates statistical significance, set at *p* < 0.05. Pearson’s Chi-squared tests were used. When >20% of cells had expected frequencies <5, Fisher’s Exact test was used.

**Table 3:** Goodness of Fit observed frequencies of racial distribution within MSK ad categories differ from US demographic distribution.

Variable	Observed Frequency; Expected Frequency				*p*-Value
	White	Hispanic	Asian	Black	
Ad Category					
Exercise	93; 79.9	10; 24.6	2; 7.9	17; 17.8	**<0.001**
Fitness/Exercise Clothing	97; 78.1	2; 24.1	3; 7.7	21; 17.4	
Fitness/Exercise Equipment	67; 53.5	2; 16.5	3; 5.3	9; 11.9	
Health Food	49; 40.3	0; 12.4	0; 4.0	17; 9.0	
Pain Relief	53; 36.7	0; 11.3	1; 3.6	4; 8.2	
Supplement	96; 70.3	2; 21.7	1; 6.9	7; 15.7	
Diet	61; 46.9	1; 14.4	0; 4.6	8; 10.5	
Health Insurance	7; 10.2	0; 3.2	0; 1.0	10; 2.3	
Weight Loss Program *	2; 4.8	0; 1.5	0; 0.5	6; 1.1	
Sports Drink	0; 25.8	0; 8.0	0; 2.5	39; 5.8	
Medical Establishment *	0; 3.0	5; 0.9	0; 0.3	0; 0.7	
Procedure *	2; 1.2	0; 0.4	0; 0.1	0; 0.3	0.728
Medical Equipment	10; 6.0	0; 1.9	0; 0.6	0; 1.3	**0.037**

* n < 10, so no significant statistical conclusions could be drawn. Bold indicates statistical significance, set at *p* < 0.05.

## Data Availability

The original data presented in the study are openly available in the RBdigital, ZINIO magazine, *Magzter,* and Amazon databases.

## Appendix A

**Genre**	**Magazines**
Elderly Health	Prevention Reader’s Digest Esquire
Women’s Health	Women’s Health Woman’s World Women’s Day
Men’s Health	Men’s Health Men’s Journal GQ
Food/Dietary Health	Tase of Home Bon Appetit Food Network
General Lifestyle	Shape The New Yorker Vogue
African American Health	Black EOE Journal Upscale Essence
Hispanic Health	Ser Padres People En Espanol Saber Vivir
Sports Health	Runner’s World Sports Illustrated ESPN the Magazine
